# Experience and results after the implementation of a radiology day unit in a reference hospital

**DOI:** 10.1186/s13244-022-01251-2

**Published:** 2022-06-29

**Authors:** Nuria Roson, Andreu Antolin, Richard Mast, Cristina Sanchéz-Tirado, Jesús Griñón, Jordi Andreu, Mercedes Perez Lafuente, Alejandro Tomasello, Manuel Escobar

**Affiliations:** 1 Department of Radiology, Institut de Diagnòstic per la Imatge (IDI), Hospital Universitari Vall d’Hebron, Passeig de la Vall d’Hebron, 119-129, 08035 Barcelona, Spain; 2 Department of Radiology, Hospital Universitari Vall d’Hebron, Passeig de la Vall d’Hebron, 119-129, 08035 Barcelona, Spain

**Keywords:** Radiology (Interventional), Patient care, Radiologists, Hospitals, Surveys and questionaries

## Abstract

**Background:**

Interventional radiological procedures have significantly increased in recent years. Most of them are minimally invasive and require a short hospitalization, mainly done in other non-radiological units nowadays. Limited bed availability and high occupancy rates in these units create longer waiting lists and cancellations. The aim of this retrospective study is to assess the creation and functioning of a Radiology Day Unit (RDU) and evaluating its outcomes. For this purpose, data about interventional procedures and its complications, incidents, patient safety, quality and satisfaction rates were collected from May 2018 to December 2020, and posteriorly analyzed to evaluate its implementation.

**Results:**

During the assessed period, 3841 patients were admitted into the RDU, with a net increase of 13% and 26% in the second and third year, respectively. Procedures performed by the Abdominal Radiology section were the most frequent (76–85%) followed by Interventional Vascular Radiology and Thoracic Radiology. Complication rates were low (1.5%) and most of them were self-limited and managed in the own department. Waiting lists were significantly reduced, from 2 months to 1 week in case of procedures performed by the Abdominal Radiology section. Patient satisfaction was higher than 80% in all the items evaluated with a global satisfaction of 93%.

**Conclusion:**

The RDU in our hospital has become a vital section for the management and post-procedure caring of patients undergoing interventional procedures in the Radiology Service with low complication rates and overall high levels of quality and patient safety, allowing the reduction of waiting lists and occupancy rates.

## Key points

- Radiology Day Unit (RDU) is a specialized unit dedicated to patient pre- and post-procedure caring.
- Fully organized by the Radiology Service staff.
- Low rate of post procedure complications and shortening of waiting lists.
- Global patient satisfaction is high (above 90%).

## Introduction

Diagnostic and therapeutic interventional procedures have significantly increased in Radiology Services in recent years. In addition, the need for biopsies is rising due to personalized oncology, especially in hospitals with a strong focus on research. These paired with recent advances in interventional radiology techniques have triggered an evolution of the specialty, from being primarily diagnostic in the past to being considered treatment units nowadays.

Most of these procedures are minimally invasive, and patients are only required to rest for a few hours before discharge. Currently, most of these patients occupy beds or spots in Day Hospitals from other medical specialties, even for elective procedures and short hospital admissions of less than 24 h. This results in longer waiting lists because of limited bed availability and high occupancy rates in other services and could also cause cancellation of the elective procedure.

An additional problem is the lack of specialized nurses in this kind of post-procedure caring in other non-radiological hospital facilities. Moreover, Radiology Services are usually located far from hospitalization areas, hindering communication between interventional radiologists and the nurses previously mentioned. This highlights the need for staff familiar with these interventional procedures and optimal post-procedure care [1].

Many of these procedures have low complication rates, which would allow for the post-procedure monitoring to be performed within the Radiology Service itself, given an adequate selection of patients, techniques [2–6] and trained staff. This will permit the early detection and treatment of these potential complications. Radiologists who perform interventional procedures, whether diagnostic or therapeutic, should be involved in the entire assistance process. They should act as both physicians and radiologists in multidisciplinary teams aimed at achieving most optimal patient care [7].

Day Hospitals dedicated to selected radiological interventional procedures that require short hospital admissions (< 24 h) are not standard in our country. There have been previous reports in other European countries such as United Kingdom [8, 9].

This study describes the methodology for implementing a Radiology Day Unit (RDU) in a leading university hospital, the advantages such a facility brings for the Radiology Service, and the results obtained in the first two years and eight months after its implementation. The indicators used for evaluating and assessing the functioning of the RDU are focused on overall and specific RDU activity, post-procedure complication rates, quality control (including patient’s satisfaction), and other positive gains such as reduction of waiting lists for specific procedures or net gain of hospital beds.

## Materials and methods

### Program implantation

The first step was to elaborate an operational plan to provide personalized and quality care at the Radiology Service for patients undergoing interventional procedures requiring less than 24 h of hospitalization [10].

This plan was reviewed and approved by the Clinical Direction of our hospital and by the clinical services that request interventional procedures. It consists of 11 main points (Table 1) aimed at providing quality care to patients by qualified personnel, creating an internal organization system that allows for optimal allocation of resources, establishing assistance circuits for patient care before, during, and after the procedure, and following up on incidents and immediate complications. Ultimately, the goal of the operational plan is to get the staff at the Radiology Service involved working as a Clinical Unit, such as any other unit at the hospital [11].

**Table 1:** Main points of the Operational Plan followed in the implementation of the Radiology Day Unit

1	Equipment
2	Human resources
3	General support services
4	Distribution of beds
5	Patient selection criteria
6	Administrative circuits
7	Selected interventional procedures
8	Assistance circuits
9	Informative documents for the patient and companion
10	Informed consent documents
11	Recommendations documents

### Characteristics and equipment of the RDU

The RDU is located in the same area as the rooms for ultrasound (US) and computed tomography (CT) guided interventionism, as well as vascular interventionism. It consists of a space with seven cubicles communicated with the nursing control area and is fully equipped for patient pre- and post-procedure care. In addition, it is equipped with six beds and a recliner chair, each with an individual light, nurse call button, oxygen outlet, vacuum system, and a chair for the companion. There is also a nurse control zone with a workstation with access to clinical history, office supplies, medications, and dressing material. Other services in the RDU include bathroom, catering service, and cleaning service.

It is open from 8 am until 9 pm during working days. There are two nurse shifts, one from 8 am to 3 pm and one from 2 to 9 pm. Moreover, there is a nursing assistant giving support from 9 am to 5 pm.

### Interventional procedures and eligibility criteria for admission in the RDU

The Abdominal Radiology, Thoracic Radiology, Vascular Interventional Radiology, and Interventional Neuroradiology decided upon the interventional procedures whose subsequent patient care should be performed at the RDU. Procedures that require short recovery time (< 24 h) with low complication rates were initially considered, and more procedures were progressively added given the good results of patient care and low complications rates once the study was started (Table 2).

**Table 2:** List of techniques suitable for post-procedure caring in the Radiology Day Unit

Radiology department	Interventional procedures	New interventional procedures
Vascular interventional	Gonadal vein embolization Sclerosis of venous and lymphatic malformations Diagnostic angiography Transjugular liver biopsy Liver manometry Vascular access repairs for dialysis Ureteral catheter placement Percutaneous nephrostomy	Removal of inferior vena cava filter Biliary catheter replacement Porth-a-Cath and dialysis catheters placement
Neuro-vascular interventional	Diagnostic cerebral arteriography	Selected arterio-venous malformation treatment
Abdominal radiology	Liver biopsy Kidney biopsy Abdominal nodule/mass biopsy Lymph node biopsy Soft tissue biopsy Thyroid alcoholization and radio frequency	Intratumorally drug injection
Thoracic radiology	Thoracic fine needle aspiration Thoracic biopsy	N/A

Patients with classes I and II of the American Society of Anesthesiologists (ASA) Physical Status Classification System [12] were admitted into the RDU. In concordance with the clinicians that request these procedures, the following patients were excluded: patients with uncontrolled diabetes mellitus, uncontrolled arterial hypertension, intake of anticoagulants or antiplatelet drugs with coagulation factor alterations not properly reverted, severe renal insufficiency or severe cardiomyopathy, and patients unable to follow instructions for subsequent care at their homes and/or without proper family support.

### RDU workflow

In coordination with clinicians and radiologists, selected staff members are in charge of scheduling interventional-guided procedures (US, CT, vascular interventionism) according to requests.

Upon arrival the patient is admitted and identified with an Identity Document (ID) wristband. An informative document about the RDU and a copy of the informed consent form is also handed to the patient. The nursing team is in charge of welcoming and accommodating the patient inside the RDU, as well as explaining the procedure and ensure proper preparation. To accelerate this process the nursing team calls the patient 48 h before the scheduled day to corroborate the following items: recommended hours of fasting, pre-procedure personal hygiene, prescribed medication (which shall be brought to the RDU and taken the day of the procedure), anticoagulation regimen, allergy history, and appointment verification. It is also recommended to get a companion the day of the procedure.

Before the procedure, the interventional radiologist introduces himself/herself and confirms that the patient understands the procedure as well as answering any questions or doubts.

Once the intervention is done, the patient is taken back to the RDU, where the nursing team will follow up on the patient and make the appropriate patient care according to the indications of the radiologist, as well as monitoring possible complications. Once discharged, the nursing team hands the patient a document containing specific post-procedure and the opening hours of the RDU, as well as a phone number in case of questions or doubts. The radiologist is the final responsible of the discharge order, as well as the main responsible of the well-being of the patient while in the RDU. If a patient is not fit to discharge it is derived for longer hospitalization in the most suitable service and management of complications.

The day after the procedure, the nursing team does a follow-up call to ensure that the patient is recovering, and no complications have appeared.

### Quality control

The administrative staff at the Radiology Service keeps a record of all the interventional procedures performed at the RDU. Other things monitored are: validated and pending of review or scheduling requests, requests pending of further tests (i.e., blood tests) and schedule changes and/or cancellations (including the cause).

Any event or complication is recorded in a specific Microsoft Excel file meant for internal usage and with limited access to the RDU staff and the members of the Quality Commission of the Radiology Service. Different protocols have been established in the event of post-procedure complications depending on their nature and severity.

The responsible interventional radiologist registers the procedure, recommendations, possible complications and subsequent actions to be taken in the radiological report and the medical history of the patient, and finally the discharge order document. RDU nurses also report relevant clinical data during the post-procedure caring in the patient medical history.

Overall, recording this information makes it possible to perform a complete quality control from an administrative and assistant perspective.

### Patient satisfaction survey

Patients are given a satisfaction survey with ten closed-ended questions with 5-point scale answers about different aspects of the Service. Two open-ended questions about positive and negative aspects and one suggestion box are also provided (Table 3). In almost three years of follow-up, there have been more than 175 answers, with 95% reliability.

**Table 3:** Mean score for each item evaluated in the Satisfaction Survey and the average score

Questions	Satisfaction
Accessibility to Day Unit Hospital	87
Attention received at the radiology department secretary	89
Waiting time since arrival at the Unit	86
Hospital Day Unit accommodation	89
Facilities conditions	83
Interventional procedure explanation or information	95
Waiting list	87
Privacy and personal data protection	88
Attention received by health personnel	99
Overall satisfaction level	93
Average	89

## Results

Between May 2018 and December 2020, 3841 patients were admitted to our hospital RDU. 752 patients were admitted between May 2018 and December 2018, 1295 in 2019, and 1794 in 2020. Thus, there was an increase of 13% and 26% in the usage of the RDU from 2018 to 2019 and 2019 to 2020, respectively.

Most of the procedures are performed by the Abdominal Radiology section, followed by the Vascular Interventionism one. Procedures encased inside the Thoracic Radiology section are sparse. This is partially explained by the reluctance of the clinicians to allow less than 24 h resting after lung biopsies, even though the complication rates for lung punctures and biopsies at our Service are well below the results published in the literature (9.5% minor complications and 2.5% major complications requiring pleural drainage due to pneumothorax or embolization for bleeding) [13–15]. There has been an increase in the number of thoracic procedures performed, from 28 (2.1%) in 2019 to 53 (3%) in 2020, and the preliminary results from 2021 keep showing a growing tendency.

**Fig. 1:**
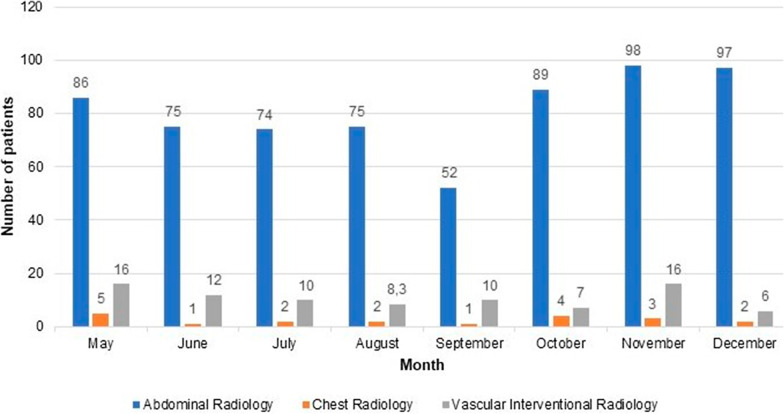
Admitted patients in the Radiology Day Unit in 2018. Number of patients admitted to the Radiology Day Unit during the last 8 months of 2018 distributed by the corresponding section

**Fig. 2:**
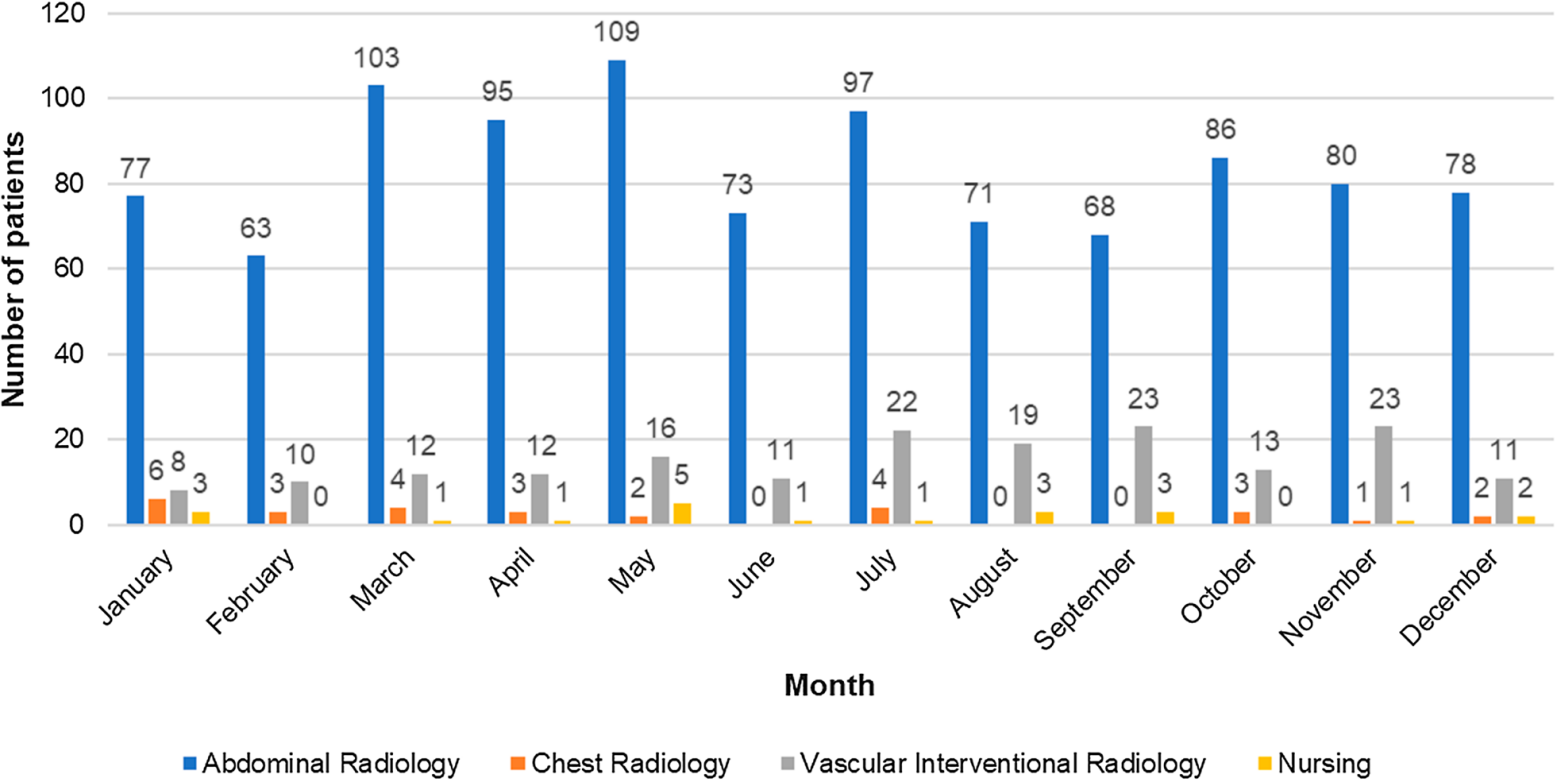
Admitted patients in the Radiology Day Unit in 2019. Number of patients admitted to the Radiology Day Unit during 2019 distributed by the corresponding section including the procedures performed exclusively by the nursing staff (i.e., CT colonography preparation)

**Fig. 3:**
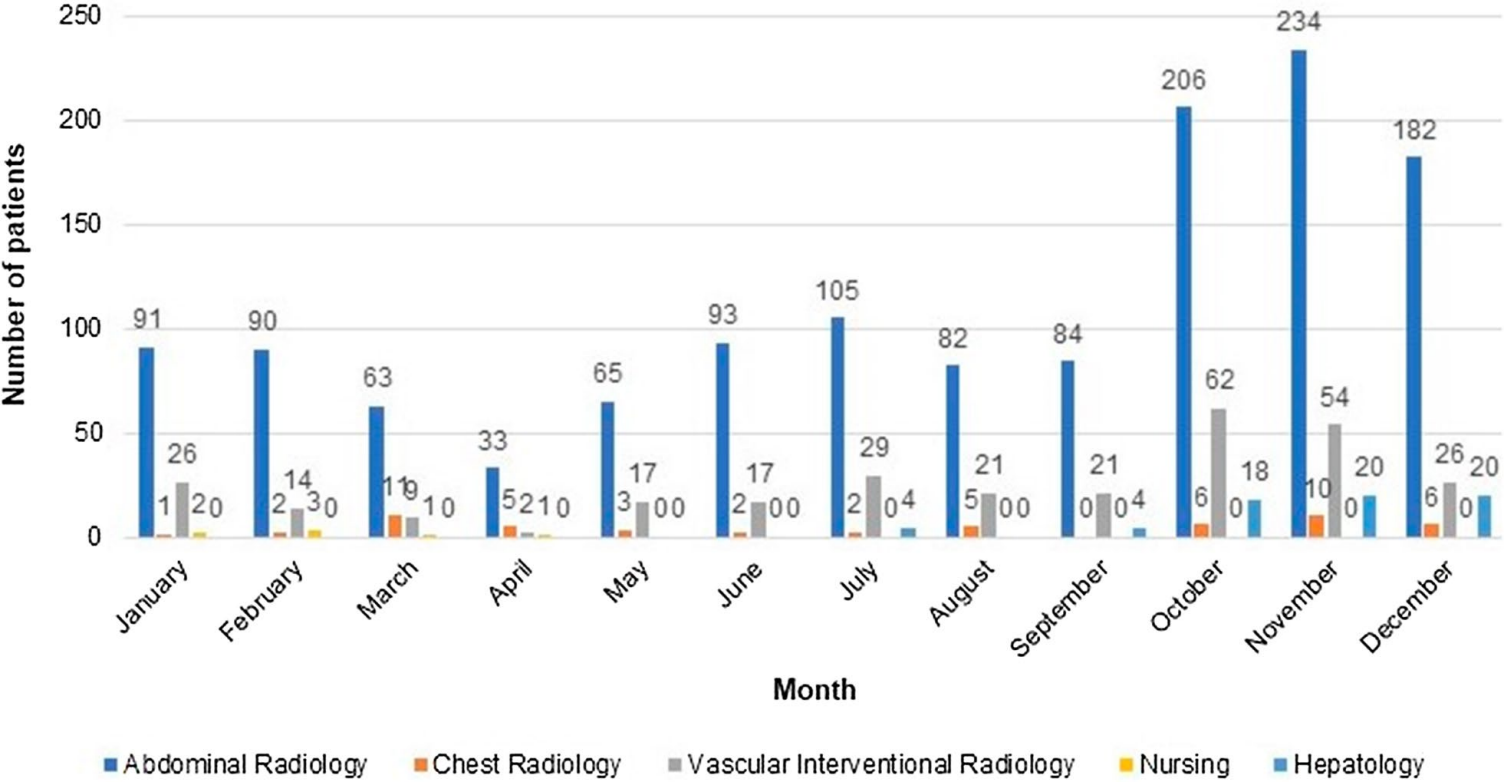
Admitted patients in the Radiology Day Unit in 2020. Number of patients admitted to the Radiology Day Unit during 2020 distributed by the corresponding section including the procedures performed exclusively by the nursing staff (i.e., CT colonography preparation) and Hepatology Service

In 2018, among the 752 patients admitted to the RDU, 642 (85%) underwent procedures performed by the Abdominal Radiology section, and 85 (11%) by the Vascular Interventional Radiology section. In 2019, out of 1295 patients, 1000 (77%) were admitted for procedures performed by the Abdominal Radiology section and 180 (14%) by the Vascular Interventional Radiology section. In 2020, out of 1747 patients, 1328 (76%) were admitted for procedures performed by the Abdominal Radiology section and 298 (17%) by the Vascular Interventional Radiology section. In 2019 and 2020, there were also procedures performed exclusively by the nursing staff such as CT colonography preparation. Moreover, from July 2020 to December 2020, there were some procedures (66 patients) performed by the Hepatology Service clinicians since their Day Unit was under construction. The lowest activity periods corresponded to summer months and intervals with a higher incidence of the COVID-19 pandemic in 2020. All this data is represented in Figs. 1, 2 and 3.

The net gain of hospital beds in other facilities due to the implementation of the RDU were 752 in 2018, 1295 in 2019 and 1794 in 2020. The waiting list for renal or hepatic biopsies performed by the Abdominal Radiology section went from two months to one week, one year after the RDU was set.

The rate of complications reported during these procedures or while the patient was resting at the RDU was 54 out of 3700 procedures performed (1.5%). Bleeding was the most common complication (30 patients, 56% of all complications), and it mainly happened in procedures performed by the Abdominal Radiology section, such as hepatic and renal biopsies. It was self-limited in 18 patients, while 12 require embolization by the Vascular Interventional radiologists. The rest of the complications are described in Table 4. There is only one case of hemoptysis, which required embolization and a prolonged hospitalization admission. None of the complications were fatal.

**Table 4:** Post-procedure complications, its frequency and treatment reported during the follow-up period at the Radiology Day Unit

Complication	Frequency (absolute/relative)	Procedure	Additional treatment
Bleeding	30 (56%)	Solid organs biopsies	Embolization (12) - Liver biopsy (6) - Kidney biopsy (5) - Suprarenal biopsy (1)
			Self-limited (18)
Arterial hypotension	9 (19%)	Kidney biopsy (1)	Clinical control or Serum therapy
		Liver biopsy (3)	
		Lung biopsy (2)	
		Thyroid fine needle aspiration (1)	
		Retroperitoneal biopsy (1)	
		Cervical node biopsy (1)	
		Nephrostomy catheter change (1)	
Pain and fever	3 (5%)	Nephrostomy (1)	Analgesic and antipyretic therapy
		Biliary catheter removal (1)	
		Liver biopsy (1)	
Pain	4 (7%)	Lung biopsy (2)	Analgesic therapy
		Liver biopsy (1)	
		Kidney biopsy (1)	
Desaturation	3 (5%)	Liver biopsy (1)	Self-limited
		Lung biopsy (2)	
Arterial hypertension	2 (4%)	Liver biopsy (2)	Self-limited
Allergy	1 (2%)	Vascular interventional procedure	Antihistaminic therapy
Contrast extravasations	1 (2%)	Vascular interventional procedure	Local treatment
Hemoptysis	1 (2%)	Lung biopsy	Embolization

The most common incident has been the lack of recent coagulation tests before the procedure. Other incidents include non-discontinuity of anticoagulant or antiplatelet drugs before procedure, lack of radiological images of target lesions in the medical history, patient misinformation and the inability to schedule the procedure in the hospital intranet.

In the particular case of the procedures performed by the Abdominal Radiology section, 48% of them reported some incident, which resulted in an overall cancellation rate of 16% in 2018 and 2019. In 2020, however, the incident rate was lowered to 22% and the cancellation rate was much lower, about 4%.

Monitoring the RDU events has allowed a constant update and improvement of the different patient-specific documents such as informed consent forms for each procedure, informative documents for patients and caregivers, post-procedure recommendations, and standard operating procedures (administrative and assistance circuits). Moreover, new records were generated as new interventional procedures were added to the RDU.

Finally, the results of the survey show a satisfaction level greater than 80% in all questions. It is worth noting that the satisfaction level for the information received about the test was 95%. The satisfaction level for the care received by the healthcare professionals was 99%, and the global satisfaction level was 93%.

## Discussion

To the best of our knowledge, there is no precedent of a similar RDU like the one explained in this article in our country, in which the Radiology Service staff is in charge of the patient before, during, and after a radiological interventional procedure. There are, however, reports of similar experiences in other countries [8, 9].

The main concern we had before the creation of this unit was patient safety. However, it is widely accepted that many of the interventional procedures in Radiology Services can be performed on an outpatient basis, given an adequate selection of procedures in low-risk patients. Several reports show that there are no statistically significant differences in the number and severity of complications between inpatient and outpatient settings for the same interventional procedures [16–20]. In our own experience, to ensure patient safety it is crucial to establish an adequate selection criterion of patients managed at the RDU. These criteria should arise from a multidisciplinary perspective, including anesthesiologists, clinicians, and radiologists. The inclusion of specialized nurses also allowed an early detection of post-procedure complications, which permitted a rapid response in the RDU itself thanks to the involvement of vascular interventional radiologists. Overall, this has resulted in low complication rates (1.5%) in the evaluated period, none of them fatal.

Patients who underwent interventional procedures at our hospital have also traditionally occupied hospital beds or spots at other Day Units. Since the creation of the RDU these procedures can still be performed in an outpatient setting in our service with similar safety levels, allowing a lower bed occupancy rate in other services. The net gaining of beds due to the implementation of RDU was 3842 over the spawn of the evaluating period. This allowed the shortening of waiting lists and reduction of cancellations, as well as avoiding longer hospitalizations in other units that does not have the RDU flexibility. This has also allowed channeling the increasing demand for biopsies of patients who are candidates for clinical trials, especially from the Oncology Service, without increasing the waiting list of biopsies needed for assistance reasons. The multi-person usage of beds during the day, doubling or tripling its use in some cases, has also contributed to the reduction of waiting lists. This is possible thanks to the coordination between the administration team and radiologists in organizing the schedule and alternating procedures that require more or less post-procedure resting time. Moreover, the armchair can be used up to five or six times during the workday because some procedures require between 30 and 60 min of resting, such as Port-a-cath implantation, superficial soft tissue biopsies, and alcoholizations or radiofrequency ablations of thyroid nodules.

The constant quality control in which the RDU was submitted also allowed the decrease in the incidence and cancellation rates in the period evaluated. This was possible thanks to the pre-48 h checklist and the constant collaboration and communication with the other clinical services to ensure that patients are fully prepared for the interventional procedure.

In the light of the above, there was an increasing usage of the RDU in our hospital during the evaluated period. The addition of tasks not directly related to an interventional procedure and performed by specialized nurses has also contributed to the rising of its usage. Some of these procedures include mild/moderate allergic reactions monitorization, treatment and control of extravasations of iodinated contrast at the CT, information about the preparation for CT colonography, and patient control before and after intratumoral injections of drugs or virus. In the second half of 2020 there was also a slight increase in its usage due to hepatic biopsies performed by the Hepatology Service since its Day Unit was under construction. The vascular interventional section has its own recovery room with 2 beds. Since the inception of the RDU, there has been a net increase in the number of vascular interventional procedures performed at the RDU due to the availability of more beds.

The 2020 COVID-19 global pandemic resulted in a decrease in the usage of the RDU, specially from March to May. This was offset in the fourth term of 2020, in which there was a peak usage of the RDU, with numbers similar to the ones that are being recorded nowadays.

The fact that the radiologist is in charge of the patient before, during, and after the procedure is a paradigm shift from a professional perspective. This was particularly surprising for radiologists at the Abdominal and Thoracic Radiology sections since they are less used to patient care and management than, for instance, vascular interventional radiologists [7, 21, 22]. Over time, radiologists who were reluctant to the opening of the RDU turned out to be its main supporters, especially because of the level of safety that comes from having the patient "in situ" with the support of specialized nursing personnel and vascular interventionism colleagues in case of severe complication. Moreover, their personal and professional satisfaction has been reinforced. These impressions have been reported informally since we have not performed an internal survey.

Other clinicians have also gained progressive trusting in the RDU. As previously stated, the number of patients that underwent thoracic procedures and rest in the RDU is increasing yearly, halving the previous reluctance of the clinicians to allow less than 24 h resting after lung biopsies. This further enhances the viability and benefits of implementing a unit of such characteristics.

The high patient satisfaction levels achieved is also an important indicator of the healthy functioning of the RDU and the benefits its implementation has offered.

There is still a long way to go for the fully implementation of these kind of units despite our results and in other countries. RDU brings benefits for patients and the hospital, since it decreases bed occupancy, shortens waiting lists and decreases cancellations, while maintaining low complication rates and high patient satisfaction. An effort shall be done to encourage the creation of this kind of units.

The main limitation of our prospective non-comparative study is the absence of data before the instauration of the RDU to make a comparison between both situations. Data before the inception of the unit was scattered and extremely hard to obtain since the patients rested in other services beds after an interventional procedure, making us unable to have control over that data.

In conclusion, the opening of the RDU at our Service has enabled the creation of a work unit in which all the professionals have common values and goals, where everybody feels that actions taken are for the patient's best interest, where we encourage sharing information and where everybody, by mutual agreement, carries out his/her work according to his/her skills and competences. This allows us to ensure patient safety with greater efficiency while decreasing bed occupancy rates and waiting lists.

## Data Availability

The datasets used and/or analyzed during the current study are available from the corresponding author on reasonable request.

## Abbreviations

ASA: American society of anesthesiologists
CT: Computed tomography
ID: Identity document
RDU: Radiology day unit
US: Ultrasound
